# Latitudinal Discontinuity in Thermal Conditions along the Nearshore of Central-Northern Chile

**DOI:** 10.1371/journal.pone.0110841

**Published:** 2014-10-21

**Authors:** Fabian J. Tapia, John L. Largier, Manuel Castillo, Evie A. Wieters, Sergio A. Navarrete

**Affiliations:** 1 Estación Costera de Investigaciones Marinas - Las Cruces, Pontificia Universidad Católica de Chile, Santiago, Chile; 2 Departamento de Oceanografía & COPAS Sur-Austral, Universidad de Concepción, Concepción, Chile; 3 Bodega Marine Laboratory, University of California Davis, Davis, California, United States of America; 4 Center for Marine Conservation - Las Cruces, Pontificia Universidad Católica de Chile, Santiago, Chile; University of Pennsylvania, United States of America

## Abstract

Over the past decade, evidence of abrupt latitudinal changes in the dynamics, structure and genetic variability of intertidal and subtidal benthic communities along central-northern Chile has been found consistently at 30–32°S. Changes in the advective and thermal environment in nearshore waters have been inferred from ecological patterns, since analyses of *in situ* physical data have thus far been missing. Here we analyze a unique set of shoreline temperature data, gathered over 4–10 years at 15 sites between 28–35°S, and combine it with satellite-derived winds and sea surface temperatures to investigate the latitudinal transition in nearshore oceanographic conditions suggested by recent ecological studies. Our results show a marked transition in thermal conditions at 30–31°S, superimposed on a broad latitudinal trend, and small-scale structures associated with cape-and-bay topography. The seasonal cycle dominated temperature variability throughout the region, but its relative importance decreased abruptly south of 30–31°S, as variability at synoptic and intra-seasonal scales became more important. The response of shoreline temperatures to meridional wind stress also changed abruptly at the transition, leading to a sharp drop in the occurrence of low-temperature waters at northern sites, and a concurrent decrease in corticated algal biomass. Together, these results suggest a limitation of nitrate availability in nearshore waters north of the transition. The localized alongshore change results from the interaction of latitudinal trends (e.g., wind stress, surface warming, inertial period) with a major headland-bay system (Punta Lengua de Vaca at 30.25°S), which juxtaposes a southern stretch of coast characterized by upwelling with a northern stretch of coast characterized by warm surface waters and stratification. This transition likely generates a number of latitude-dependent controls on ecological processes in the nearshore that can explain species-specific effects, and add strength to the suggestion of an oceanography-driven, major spatial transition in coastal communities at 30–31°S.

## Introduction

Marine environments associated with the Humboldt Current System (HCS) are among the most productive in the world [1]. As other eastern boundary ecosystems, large-scale circulation in the HCS is characterized by an equatorward geostrophic flow and coastal waters are characterized by wind-forced offshore transport of surface waters that results in the upwelling of cold, nutrient-rich waters and alongshore coastal jets. Although major changes in large-scale circulation are observed along the entire region between 20–40°S [2]–[4], a latitudinal pattern in the intensity of upwelling-favorable winds, with generally weaker but more persistent winds north of about 30–32°S and stronger but temporally more variable winds to the south, has been highlighted in early studies [1], [5], [6] and is described in detail in a recent review of near surface winds in the South Pacific [7]. Latitudinal variation in satellite-derived surface chlorophyll occurring at about 30–32°S has been attributed to these regional changes in wind patterns [8]–[11]. However, the consequences of this latitudinal shift in wind regimes on the physics of the coastal ocean, i.e. its circulation and hydrographic structure, have been little investigated. Focusing on the connection between the coastal and offshore ocean, Hormazábal *et al.* [12] examined 7 years of subsurface current observations from offshore moorings together with satellite data on sea level anomaly and wind stress, to find that eddy kinetic energy is enhanced in the region of strong but variable winds south of ∼30°S, and weak north of this latitude. Other studies have used the high resolution atmospheric model implemented by Falvey *et al.* [13] to drive a circulation model for central Chile and project changes for different climate scenarios [14]. Yet, none of the above work provides insight on whether latitudinal changes are to be expected for hydrodynamic and hydrographic conditions in nearshore waters.

We are specifically interested in the dynamics over the inner shelf that may connect regional patterns in offshore circulation and in the wind field to spatial patterns nearshore and along the shoreline. This is where a large fraction of biological productivity and diversity are found, and where many fisheries for invertebrates, fish and algae are concentrated. In north-central Chile, a number of ecological studies along the shoreline have documented major spatial shifts in pelagic and benthic communities, suggesting the existence of a transition in the physical environment nearshore. Specifically around 32°S, major changes have been observed in species composition [15], [16], in the abundance of dominant macro-algal groups and intertidal mussels [15], [17], and in the intertidal rates of recruitment of several sessile invertebrate species [10], [18]. It has been suggested that changes in upwelling regimes, especially the frequency of upwelling relaxation events, drive the dynamics of onshore delivery of benthic invertebrate larvae, leading to an extensive region of low onshore recruitment and generally low population sizes north of this latitude [10]. Latitudinal shifts in physical variability may also underlie observed genetic structure in intertidal barnacle and macroalgal species [19]–[21]. A recent review concludes that genetic structure in eight invertebrate species is linked to their dispersal potential as well as to historical differences in nearshore environmental conditions north and south of 30°S [22]. Thus, biological evidence points toward the existence of major changes in nearshore habitats in a transition region between 30 and 32°S.

Despite these potentially major and varied consequences for the biology of nearshore pelagic and benthic communities, latitudinal changes in the nearshore physical environment of the HCS remains poorly characterized. To date, field studies have been local and emphasized patterns in thermal structure and circulation associated with smaller scale variation in topography, coastline orientation and freshwater inputs [23]–[30]. It is therefore an open question whether a latitude-dependent pattern can be identified over hundreds of kilometers in north-central Chile that can potentially explain the observed patterns in ecological phenomena. Here we describe a latitudinal transition in thermal conditions based on a set of shoreline temperatures collected over 10 years at 15 sites in central Chile between 28 and 35°S. Thus, our primary data represent the environmental conditions and regimes of thermal variability actually experienced by the benthic organisms that exhibit significant population changes around 30–32°S. We complement these *in situ* observations with satellite-derived temperature imagery to examine the offshore extent of spatial structures, and with satellite-derived wind information to identify alongshore structure of upwelling forces. We identify a marked transition in nearshore thermal conditions centered at 30.25°S and ascribe it to a latitudinal transition that is sharpened by sudden changes in winds and topography in this region and combined with a sharp cape-to-bay transition. Further, by using temperature data as a proxy for nutrient concentrations, we can explain a notable transition in the biomass of corticated algae at this latitude – providing an explicit example of how the well-marked transition in nearshore thermal conditions may drive a collection of ecologically important transitions at this latitude.

## Materials and Methods

### Data collection

**Figure 1:**
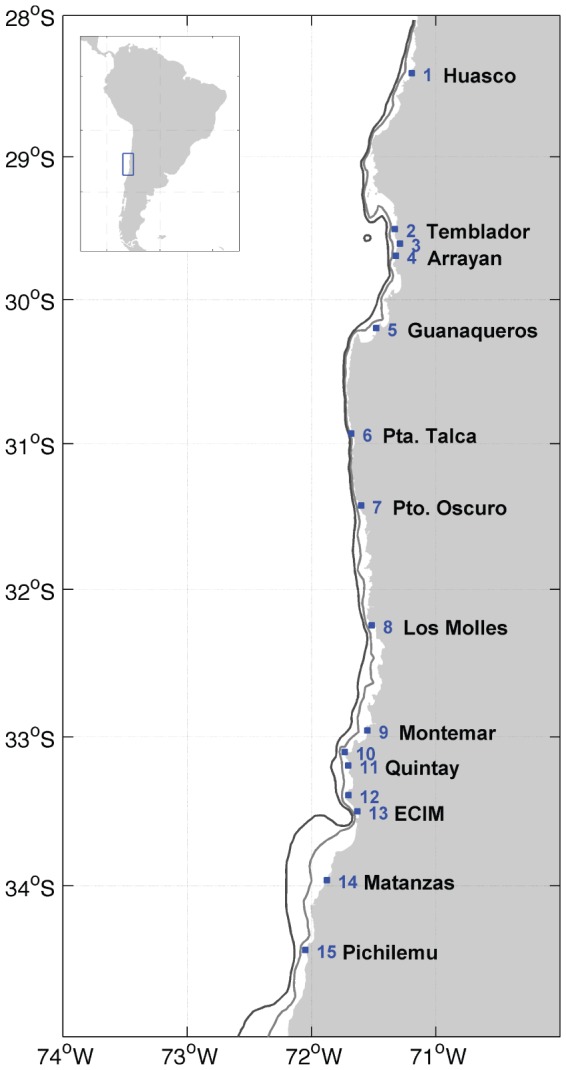
Location of the 15 study sites in central-northern Chile (see also Table 1). Contours on the map correspond to the 100 and 200 m isobaths.

Between 28°24′S and 34°25′S, we measured water temperature at 15 sites (Fig. 1, Table 1) using Stowaway TidBit loggers (Onset Computer Corp., USA) deployed in shallow-subtidal waters, approximately 1 m below Mean Lower Low Water (MLLW). Loggers were programmed to record instantaneous temperatures with sample intervals that ranged from 5 to 10 min, and maintained for periods of 4 to 10 years at different sites (see Table 1). No specific permissions were required to conduct measurements reported here. Field measurements did not involve endangered or protected species. Specific locations of measurement sites are shown on Table 1.

**Table 1:** Names and locations of coastal sites, together with descriptive statistics of temperature data sets used in the analysis.

Site		Latitude	Longitude	Mean + SD			Median	Max.	Min.	Dates			N obs.	Gaps
#	Name	[S]	[W]	[°C]			[°C]	[°C]	[°C]	Begin		End	[d]	[%]
1	Huasco	28°24'	71°11'	14.73	±	1.40	14.41	20.05	11.89	5-Sep-02	-	6-Jan-08	1950	1.8
2	Temblador	29°30'	71°19'	14.15	±	1.53	13.71	18.80	11.54	7-Aug-01	-	10-Mar-08	2408	16.1
3	Cta. Hornos	29°39'	71°19'	14.25	±	1.62	14.02	19.33	11.11	1-Dec-02	-	22-Jan-08	1879	1.6
4	Arrayan	29°41'	71°19'	14.20	±	1.44	13.90	18.82	10.78	9-Sep-99	-	9-Mar-08	3105	0.0
5	Guanaqueros	30°11'	71°28'	14.63	±	1.61	14.29	18.77	11.44	12-Jun-02	-	7-Mar-08	2096	10.4
6	Pta. Talca	30°55'	71°40'	13.25	±	1.28	13.03	18.40	10.39	6-Feb-01	-	10-Dec-07	2499	6.2
7	Pto. Oscuro	31°30'	71°36'	12.80	±	1.36	12.60	18.30	9.82	16-Sep-04	-	4-Apr-08	1297	1.9
8	Los Molles	32°14'	71°30'	13.62	±	1.57	13.27	19.50	10.28	31-Dec-00	-	11-Oct-07	2476	5.0
9	Montemar	32°57'	71°32'	13.19	±	1.18	12.94	17.60	10.97	13-Mar-02	-	4-Jan-07	1759	0.0
10	Curaumilla	33°06'	71°44'	12.61	±	1.36	12.31	18.05	10.30	10-Dec-01	-	26-Sep-07	2117	6.3
11	Quintay	33°11'	71°42'	13.30	±	1.34	13.05	19.19	10.53	24-Apr-98	-	23-Oct-07	3470	13.1
12	El Quisco	33°23'	71°42'	13.02	±	1.22	12.78	18.12	10.93	30-Mar-02	-	5-Jan-07	1743	14.1
13	ECIM	33°30'	71°37'	13.64	±	1.66	13.22	20.15	10.80	21-Feb-01	-	8-Mar-08	2573	7.0
14	Matanzas	33°57'	71°52'	13.06	±	1.24	12.83	18.88	10.69	5-Sep-97	-	25-Aug-07	3642	12.7
15	Pichilemu	34°25'	72°02'	12.95	±	1.21	12.68	18.17	10.73	1-Jan-01	-	20-Apr-08	2667	20.5

Data on wind variability near each site were obtained from daily QuikSCAT wind fields measured over a 0.25° resolution grid for the period July 1999 through October 2009 (ftp://podaac.jpl.nasa.gov/pub/ocean_wind/quikscat). Although QuikSCAT wind measurements are considered reliable for distances ≥25 km from shore [31], we increased such minimum distance to 50 km to ensure data reliability and to minimize temporal gaps in the data series. *In situ* wind data were obtained from the weather station of the Estación Costera de Investigaciones Marinas (ECIM) at Las Cruces (33°30′S, 71°38′W), which recorded wind speed and direction at 20 min intervals, and used to assess whether QuikSCAT velocities 50 km offshore are representative of winds measured on the coast. Over the period 2000–2004, daily averages of the *in situ* wind record and QuikSCAT-derived winds showed high coherence (*r*>0.8) for periods greater than 5 days, with approximately zero phase lag; this coherence is even higher during spring-summer periods (Fig. S1).

To assess the offshore extension of the alongshore thermal structure, we used Level-3 SST images (8-day composites, 4×4 km resolution) from the MODerate-resolution Imaging Spectroradiometer (MODIS-Aqua) for the period between January 2003 and March 2013. The satellite-derived time series for SST at 5 km offshore showed a reasonable coherence with daily *in situ* data, with zero-lag correlation coefficients ranging from *r* = 0.77 to *r* = 0.94 (*p*<0.001, see Fig. S2).

To explore potential biological consequences of thermal variability for ecologically important members of rocky intertidal communities, we estimated biomass of corticated macroalgae, which frequently dominate the low intertidal zone [15], [17] and appear to be strongly influenced by changes in upwelling regimes [17], [32], [33]. Quantitative field surveys were conducted at similarly wave-exposed platforms at 13 of the study sites spread between 29.5° and 34.5° S during austral spring and summer months between 2001 and 2004, with all sites surveyed in more than one year. At each site, percentage cover of macroalgal species was estimated within a minimum of eight 0.25 m^2^ quadrats haphazardly placed along transects stretched parallel to shore across the low intertidal zones of 2–3 rocky benches. Biomass (wet weight) per species was calculated for each quadrat using area/mass regressions. Site-specific conversion equations for all dominant species (>5% cover) were obtained by collecting a minimum of 5 samples per species from each site. Species abundances were pooled into pre-defined functional groups [15], [17]. Finally, mean biomass (g m^−2^) estimates presented here were obtained by pooling replicate benches and years for each site.

### Data analysis

Raw *in situ* temperature data were quality-controlled and low-pass filtered (40-h Lanczos-cosine filter) to remove diurnal, tidal, and inertial-scale variability, including high-frequency fluctuations such as internal tidal bores [34]. Analysis of daily averages produced from these filtered temperature time series is the primary component of this investigation. Short gaps in the records (<5 days) were filled using linear interpolation. We calculated descriptive statistics, seasonal means, and climatologies of daily temperature for each site.

To quantify the relative importance of synoptic, intra-seasonal and seasonal variability in daily temperatures, we conducted spectral analyses using Welch's modified average periodogram. For statistical reliability of the spectral estimates, each time series was divided into segments of 366 d with a 50% overlap between adjacent segments. The resulting spectra were then averaged to obtain a representative spectrum for each site [35]. Relative contributions to total spectral density (TSD) were then calculated for a “seasonal band” (periods of 300–100 days), an “intra-seasonal band” (100–20 days) and a “synoptic band” (20–2 days). Additionally, an empirical orthogonal function (EOF) analysis was performed for all gap-free temperature time series over a 4-year period (16 September 2004 to 5 June 2008).

To characterize the offshore scale of latitudinal patterns in sea surface temperature variability, harmonic and EOF analyses were performed on SST fields derived from Level-3 MODIS-Aqua imagery for the region defined by 28–35°S and 71–74°W. From each 4×4 km pixel within this region that contained good-quality data (i.e. valid data in more than 20% of the images), we extracted a daily time series of temperature and fitted an annual harmonic signal (i.e. period 365.25 d) using the least-square procedure described by Emery & Thomson [35]. Amplitude of the fitted annual harmonic, as well as residual variance (i.e. fraction of total variance in the time series that was not explained by annual harmonics) were calculated for each pixel over the region. Finally, an EOF analysis was performed on a set of 8-day SST composites over the same region. This time-averaging was done in order to reduce the fraction of each time series – from individual pixels – that corresponded to gaps. EOFs were computed using only those pixels that contained valid data in at least 70% of the composite images.

The relationship between *in situ* temperature variability and coastal wind stress was examined with cross-correlation analyses. We used daily time series of meridional wind stress from the QuikSCAT pixel closest to each study site and daily in situ temperature anomalies. The latter were calculated by subtracting a 30-day running mean from each daily time series of temperature averages, which is an effective way to remove longer time scales of variability (i.e. seasonal and intra-seasonal), as well as the confounding influence of latitudinal differences in mean water temperature [30]. Finally, to assess latitudinal patterns in thermal variability that might indicate changes in bottom-up forcing on coastal benthic communities [36], we computed the fraction of spring-summer days with in situ temperatures at or below 12°, 13° and 14°C. This choice of values was based on the negative linear relationships between temperature and nitrate concentration documented for inner-shelf waters in central Chile [32], [33], with ∼30 uM nitrate at 12°C and near-zero nitrate concentrations above 14°C. We used this value as a threshold indicating favorable and unfavorable conditions for the growth of coastal micro- and macro-algae. We expect that an onshore benthic community at sites with a greater fraction of days below 14°, 13° and 12°C faced increased nutrient regimes and potentially higher primary productivity.

## Results

### Thermal variability

**Figure 2:**
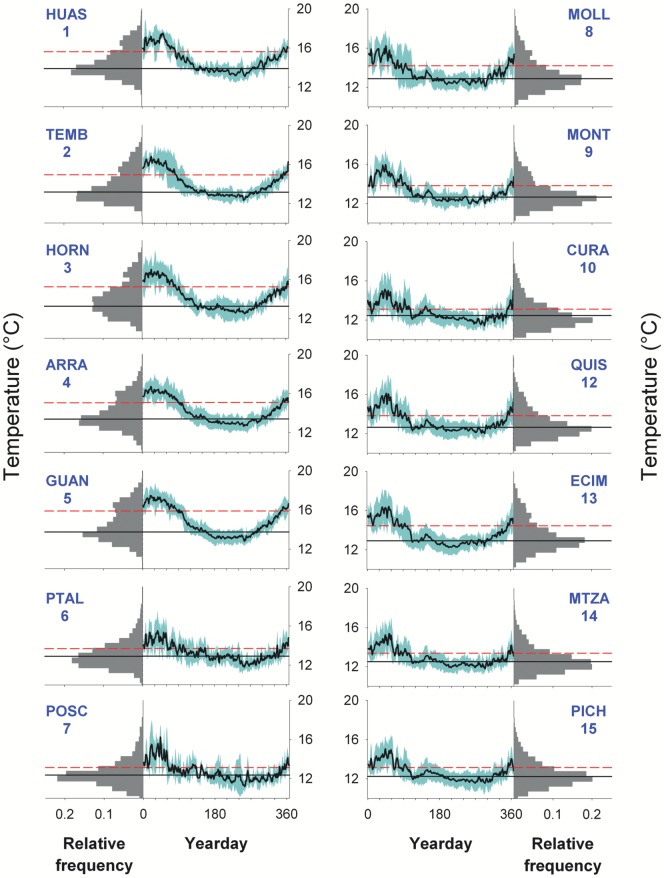
Statistical description of *in situ* temperature records at 14 of the study sites. Bar and time-series graphs correspond to frequency distributions and climatologies of water temperature, respectively. Shaded areas around climatologies represent interannual variability (±1SD). Solid and dashed horizontal lines correspond to fall-winter and spring-summer mean values, respectively. Numbers and four-letter acronyms on each panel correspond to site numbers and names (see Fig. 1 and Table 1).

A coherent and marked seasonal pattern of temperature was observed throughout the study region between 28 and 35°S (Fig. 2), with warmest water in late summer and coolest water in winter. In the north (sites 11–15), temperatures exhibited a bimodal distribution separating typical winter temperatures ∼13–14°C from typical summer temperatures ∼15–16°C, whereas in the south distributions were uni-modal, long-tailed and cooler. Coldest mean temperatures, and the smallest differences between summer and winter means, were observed at headland sites that are upwelling centers: sites 14 and 15 at Matanzas/Pichilemu, site 10 at Curaumilla, and sites 6–7 at Punta Talca and Puerto Oscuro (Table 1, Fig. 1–2). Sites in bays exhibited higher mean temperatures than sites nearby (e.g., site 13 at ECIM and site 5 at Guanaqueros). In addition to this small-scale spatial pattern, there was a marked latitudinal trend, with increasing temperatures to the north (Table 1, Fig. 2).

**Figure 3:**
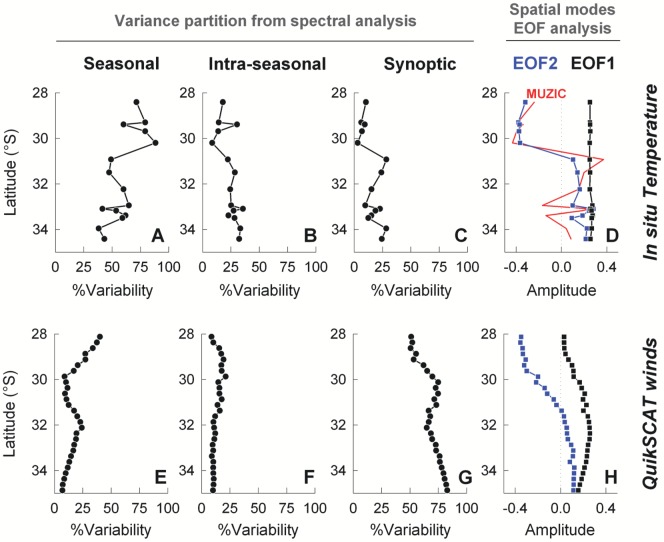
Latitudinal patterns in the variability of *in situ* temperature (A-E) and QuikSCAT alongshore wind (F-J). Panels on the left (A, F) show latitudinal long-term means for each variable. Panels on the mid three columns show percentages of total variability derived from spectral analyses that correspond to annual (B, G), intraseasonal (C, H), and synoptic (D, I) fluctuations. Panels on the far right (E, J) show the latitudinal distribution of the first 2 modes of EOF analyses. Red line in (E) corresponds to latitudinal distribution of the MUZIC index of upwelling-induced variability [30].

Notable differences in the shape of the seasonal temperature cycle were observed (Fig. 2): in the south, a long cool season (May to November) is followed by a short warm season (January to March), whereas in the north the thermal cycle is more sinusoidal with warming starting in September. A sudden cooling in late summer (around day 110) and a brief warm period in fall (roughly days 140–170) are common to many southern sites. Although an examination of the daily time series indicated that events like these occurred more than once and at several sites, they likely do not represent a true climatology. Spectra of temperature were dominated by a strong seasonal peak, explaining ∼75% of the variance in temperature at northern sites and ∼50% of the variance at southern sites (Fig. 3). In the south, higher frequency variability was more evident, with both intra-seasonal and synoptic-scale variability each explaining ∼25% of the variance (Fig. 3). Between 30 and 31°S, a sharp transition in the relative importance of seasonal and synoptic variability was observed (Fig. 3a-c). The latitudinal decrease in synoptic variability was also well captured by a multivariate index of upwelling-forced cooling (MUZIC, Fig. 3d), which is based on features of the cooling events that occur during the spring-summer upwelling season [30].

An EOF of the 15 time-series of temperature yielded a first mode that explained 65% of the variance (EOF1) and a second mode that explained 15% of the variance (EOF2, Fig. 3d). The first mode exhibited uniform latitudinal structure and a time-dependence that is markedly seasonal (Fig. S2b) – this is consistent with the dominance of the seasonal signal in all individual temperature records (Fig. 2). On the other hand, the sharp latitudinal step in the spatial structure of EOF2 (Fig. 3d) reflected an abrupt spatial change in the amplitude of variability between 30 and 31°S. EOF2 was characterized by intra-seasonal and synoptic-scale frequencies, with variance similar to EOF1 for periods shorter than 100d (Fig. S2b-c).

**Figure 4:**
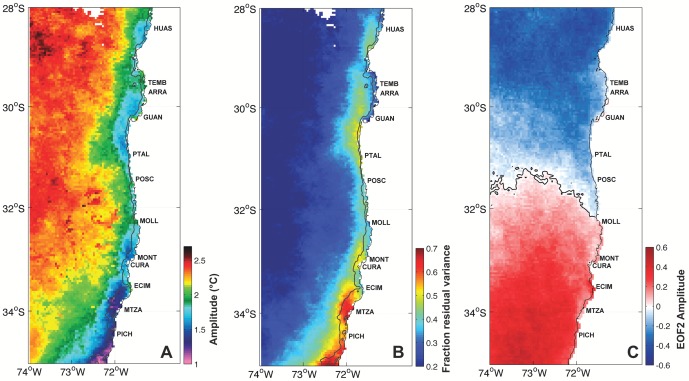
Spatial pattern in SST variability derived from harmonic analysis (A, B) and EOF analysis (C) of MODIS-Aqua imagery for 2003–2013. (A) Amplitude of the seasonal cycle. (B) Residual variance, i.e. not explained by the seasonal cycle. (C) Spatial pattern of the second mode of variability from the EOF analysis of weekly SST composites.

Satellite-derived time series of SST in coastal waters also exhibited strong seasonality, with the amplitude of the seasonal signal nearshore increasing from 1°C in the south to 2°C in the north – and up to 3°C offshore, beyond the coastal band affected by coastal upwelling (Fig. 4a). Higher frequency variability accounts for ∼60% variance in the south, decreasing to ∼30% in the north (Fig. 4b). Although constructed from a broad swath of offshore data, the EOF2 for SST also shows a north-south transition, located at 31–32°S (Fig. 4c).

### Wind variability

In contrast with temperature variability nearshore, meridional wind stress at 50 km offshore did not show such marked latitudinal changes – however, there is an increase in the proportion of variance due to synoptic variability around 30°S (Fig. 3e,f,g). The first EOF mode for wind data explained 65% of total variance with little latitude dependence, whereas the second EOF explained 22% of total variance, with greatest latitude-dependent change between 30 and 31°S (Fig. 3h). Both modes exhibited strong seasonal signals and synoptic variability, with weak inter-annual variability (Fig. S2e-f).

#### Wind-temperature correlations

**Figure 5:**
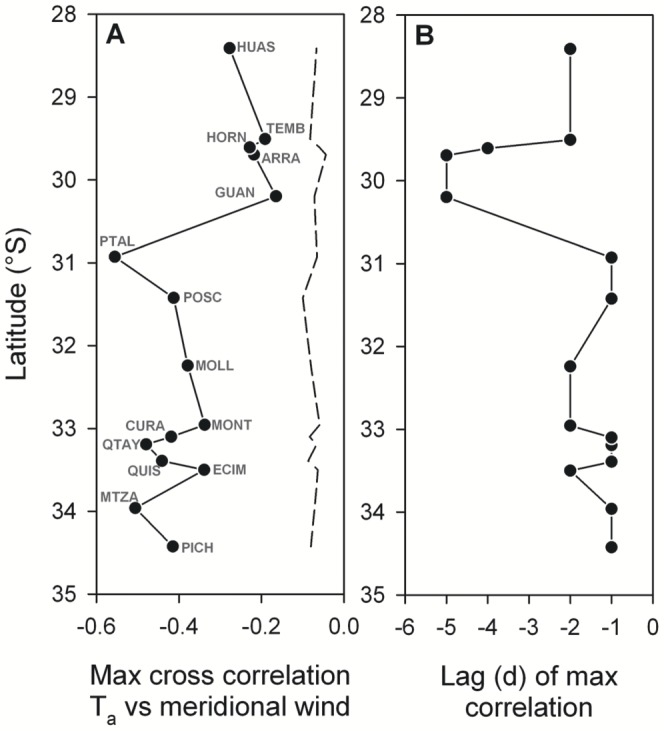
Latitudinal pattern in thermal response to wind variability. (A) Maximum cross-correlation between daily *in situ* temperature anomalies and alongshore wind stress, (B) time lag (days) corresponding to the maximum cross-correlation. Dashed line in (A) indicates the 95% significance limit for each site-specific correlation.

Correlations between wind stress and *in situ* temperature variability showed that stronger equatorward winds led to significant drops in nearshore temperature at all sites across the region (Fig. 5a). In general, sites located in/near upwelling centers exhibited strongest negative correlations (e.g., PTAL, QTAY, MTZA) with thermal response lagging wind forcing by 1 day (Fig. 5b). All sites south of 30.5°S exhibited stronger correlations (0.35–0.55) than sites north of this latitude (0.20–0.35). For all latitudes, a slower thermal response was observed at more sheltered sites located in bays and/or downstream of upwelling centers, e.g. ECIM, MONT, and MOLL with a 2-day lag, and sites in the most sheltered part of the larger Coquimbo - La Serena Bay (GUAN, ARRA) with a 5-day lag that suggests enhanced retention of nearshore waters.

### Thermal patterns and algal biomass

**Figure 6:**
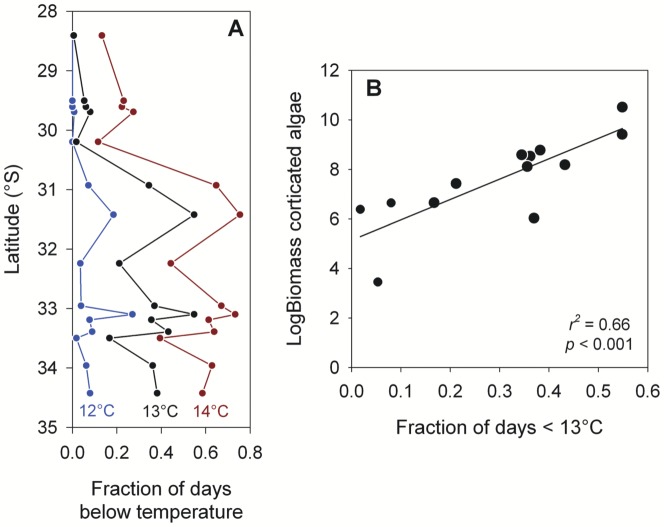
Spatial pattern in nearshore cooling and its relationship with algal biomass. (A) Latitudinal pattern in the summertime occurrence of temperature conditions indicative of high (12°C) to limiting (13°C) nitrate supply to shoreline habitats. (B) Association between cooling frequency (<13°C) and the biomass of corticated algae at a subset of the sites for which temperature records are available.

Noting warmer temperatures and a weaker response to upwelling winds at northern sites, one would expect colder, nutrient-rich water to be less common in these nearshore environments. Based on prior work that has shown a robust temperature-nitrate relationship in shallow-subtidal environments of central Chile [32], [33], for each site we calculated the probability of exposure to water <12°C, <13°C or <14°C, which represent high levels (∼30 µM), moderate levels (∼17 µM), and low levels (∼0 µM) of nitrate, respectively. On average, sites located south of 30.5°S were exposed to temperatures equal to or less than 14°, 13° and 12°C for 61%, 37% and 9% of the spring-summer season, respectively (Fig. 6a). Cold, nutrient-rich water was most frequently observed at shoreline sites located at or near upwelling centers (e.g. Curaumilla, Puerto Oscuro) and least common at sites located downstream or in upwelling shadows such as ECIM and Los Molles (Fig. 6a, and see Fig. 1). North of 30.5°S, sites were seldom exposed to waters <12°C and rarely experienced waters <13°C or <14°C (less than 10% or 20% of summer days, respectively). These spatial differences in the exposure of nearshore sites to cold water were correlated with differences in the biomass of corticated algae at these sites (Fig. 6b), consistent with the idea that exposure to low temperatures is a proxy for availability of nitrate, which is a limiting nutrient for the growth of corticated algae. Sites with more frequent cold-water events exhibited greater algal biomass than those where these events are less frequent. Thus, the differences in thermal conditions we have observed across the 30–31°S transition may exert control on ecological phenomena both directly and through their influence on nutrient availability.

## Discussion

We have analyzed a unique set of shoreline temperature data to investigate the apparent latitudinal transition in nearshore oceanographic conditions suggested by recent ecological studies along central-northern Chile. Evidence of abrupt latitudinal changes in the dynamics, structure and genetic variability of benthic communities along the Chilean coast has been found consistently between 30° and 32°S [10], [21], [22], [37]. While alongshore changes in the advective and thermal environment have been inferred from ecological patterns [10], [15], [17], [37], an analysis of *in situ* physical data has been missing. In our results a marked transition in thermal conditions is observed between 30° and 31°, superimposed on a broad latitudinal trend and small-scale structures associated with cape-and-bay topography.

A stronger seasonal signal in shoreline thermal variability north of 30–31°S is apparent in the climatology and the bi-modal distributions of temperature values (Fig. 2), as well as in the percentage variance in the seasonal band (Fig. 3). This transition in seasonality overlays a general latitudinal trend towards warmer mean annual temperatures north of 31°S, specifically in summer values and in the winter-summer difference. More generally, in coastal waters (satellite data, Fig. 4), the amplitude of the seasonal signal increases from 1 to 2°C moving north, with weakening of this signal at well-defined upwelling centers (Matanzas 34°S, Curaumilla 33°S, Punta Talca 31°S), and strengthening inshore downstream of upwelling centers (Las Cruces at 33.5°S, Los Molles at 32.5°S, Guanaqueros at 30°S, and the northern half of the Coquimbo – La Serena Bay at ∼29.5°S). In the south and specifically in the vicinity of upwelling centers, summer and winter temperatures are actually quite similar on average due to the intensification of upwelling-driven coastal cooling in summer.

In contrast, the synoptic signal in shoreline thermistor data is relatively strong south of the 30–31°S transition, as shown by the spread of values comprising climatologies (Fig. 2) and by the percentage of variance in the synoptic band (Fig. 3). This is also evident in coastal waters, as seen in satellite data (Fig. 4b), with more than half of the variability being non-seasonal south of the 30–31°S transition (mostly synoptic), whereas non-seasonal variability contributes only a third in nearshore waters north of the transition. This pattern in thermal variability derived from both *in situ* temperatures and satellite SST is consistent with a drop in the percentage of wind variability that corresponds to the synoptic band (Fig. 3) north of 31°S.

The north-south transition in thermal conditions is well represented by an EOF analysis, in which the first mode describes a coast-wide seasonal signal with some intra-seasonal variability while the second mode exhibits a marked switch at 30–31°S. This EOF2 thus captures the notable transition north and south of 30–31°S. Seasonal variance in EOF2 is weak, but synoptic variance is as strong as in EOF1 and intra-seasonal variance is weaker but similar to that for EOF1 (Fig. S2). The temporal structure of EOF2 shows that peaks in summer are of opposite sign to peaks in EOF1, so that north of the transition synoptic/intra-seasonal variability is muted (smoother seasonal cycle) and south of the transition this higher frequency variance is enhanced (Fig. S2). In this way, EOF2 also differentiates between seasonality with a prolonged warm-water period in the north (December-March) and a brief warm period in the south (February-March). The remarkably similar trend between EOF2 and the Multivariate Upwelling Zone Index of Cooling (MUZIC) index calculated for each site, which is designed to capture upwelling-induced variability and more specifically the synoptic-scale cooling events produced by coastal upwelling in spring-summer months [30], reinforces the importance of synoptic variability associated with upwelling winds in the second EOF computed here for *in situ* temperature series. A similar north-south pattern is seen in the second EOF of satellite SST data, with a change in sign at about 32°S near the shore (Fig. 4c). Probably because this analysis was primarily comprised of SST data from offshore waters, the latitudinal pattern does not appear as dramatic as the transition detected at the shoreline with *in situ* temperatures. Such difference highlights the potential role of shoreline configuration and topography as enhancers of latitudinal trends in the extent to which larger-scale oceanographic variability is perceived in littoral environments.

Consistent with this last statement is the marked transition detected between 30 and 31°S in the response of shoreline temperatures to wind forcing (Fig. 5); while still significant north of the transition, the correlation is notably weaker. Furthermore, thermal response time becomes substantially longer at sites Guanaqueros and Arrayan (5 days), suggesting local retention effects at some sites in the Coquimbo – La Serena bay. This bay effect is not apparent at sites locates further north, such as Temblador and Huasco, where the time lag of thermal response is comparable with that at sites elsewhere (Fig. 5). This latitudinal transition in the response of temperature to wind variability is also well captured by the MUZIC index. Surface warming and stratification of nearshore waters are expected to increase to the north, consistent with an increase in surface heat flux and a drop-off in coastal wind forcing north of Punta Lengua de Vaca [7]. Such changes are likely to induce a transition in the balance between potential energy from stratification and kinetic energy from wind forcing, which controls whether sub-thermocline waters break the surface at the coast or not.

Underlying the observed transition in thermal conditions are latitudinal changes in winds and topography. Rahn and Garreaud [7] describe a maximum in southerly winds along the coast, extending from about 40°S (just south of Punta Lavapie) to about 27°S (just north of Punta Lengua de Vaca) and a maximum in wind seasonality at the southern end of this range that drops to about zero seasonality at ∼30°S. Thus, it appears that in the southern part of our study region seasonal upwelling counter-balances seasonal changes in surface heating, yielding low temperatures all year. Upwelling winds are less seasonal in the northern part, so that seasonality in surface warming yields seasonality in shoreline temperatures. Further, as noted above, a critical balance between surface warming and wind stress appears to be attained in the north, such that upwelling events do not always result in the thermocline breaking the surface near the coast. Although not explored here, there is also a significant increase in the importance of diurnal variability at northern sites. Both Muñoz [38] and Rahn and Garreaud [7] show a maximum in diurnal winds between 20° and 30°S, which occurs as a diurnal acceleration of alongshore winds. We see a similar enhancement of diurnal temperature variability in shoreline temperature data (unpublished results), which may also result from the potential for resonance of diurnal winds with inertial period at 30° latitude [39] and from the presence of diurnal internal tides only at lower latitudes, i.e. where the inertial period is >24 hours.

In addition to the increased height and decreased distance to the coast of mountains north of 30°S, which influence large-scale wind patterns [7], [38], the presence of a convex coast with elevated topography at Punta Lengua de Vaca and enhanced gradients between cold ocean and warm land just north of the point result in a local wind maximum here [7], [40]. Further, the shelf between 30.5 and 31.5°S is extremely narrow (Fig. 1), which allows for upwelling of deeper waters and reduced nearshore retention of upwelled waters such that temperatures are likely to be coldest here. Indeed, minimum temperatures are observed at Punta Talca and Puerto Oscuro (Figs. 2,3).

In this work we advance the characterization of nearshore thermal conditions along the Chilean coast, building on prior studies that recognize lower temperatures at upwelling sites such as Matanzas, Curaumilla, and Punta Talca [23], [24], [41] and higher temperatures at sites in bays such as at Las Cruces [26], [28], Montemar and Guanaqueros [32]. This topographically induced mesoscale structure is superimposed on a latitudinal trend in upwelling-driven thermal variability [30]. Also, this work corroborates recent satellite-based studies that show a latitudinal transition in the relative importance of the seasonal SST cycle near 30–32°S [17], [42] and a number of studies that show how nearshore temperatures are driven by synoptic variability in coastal winds [23], [43]–[45]. Additional influences with strong latitudinal trends may come from offshore. For example, coastally-trapped waves have been identified as a source of temperature variability in the coastal ocean off Chile [46], [47]. Further, Hormazábal *et al*. [12] show a distinct offshore zone of enhanced mesoscale eddy energy between 30° and 40°S. While these offshore dynamics may influence the inner shelf [48], this is likely to be a secondary factor contributing to the nearshore latitudinal transition in thermal conditions.

Our understanding is that the marked transition in shoreline thermal conditions between 31° and 30°S stems from latitudinal gradients in atmospheric forcing (i.e. surface wind stress and heating) that are enhanced by marked changes in topography (i.e. mountains and shelf width), combined with the juxtaposition of a major upwelling center and embayment (each extending over ∼100 km alongshore). The sudden transition from the upwelling maximum between 31.5° and 30.25°S (characteristic of the south) to the warmer bay-influenced shoreline and nearshore stratification between 30.25° and 29.5°S (characteristic of the north) sharpens an otherwise gradual transition into a step-function transition between nearshore thermal conditions north and south of 30.25°S (Punta Lengua de Vaca). Beyond the bay effects towards the north (i.e. north of 29°S), conditions are distinctly northern and very different to those beyond the headland effects to the south (south of 32.5°S), where conditions are distinctly southern. Offshore, the transition is less sharp and also shifted north by the upwelling plume that extends equatorward from the upwelling center at Punta Lengua de Vaca to a latitude of about 29.5°S (satellite data, see Fig. 4b).

The marked transition in nearshore thermal conditions indicates a sharp latitudinal change in ecologically important habitat and dispersal factors. In prior studies of upwelling-driven physical and biological patterns, variability in water temperature has been used as an proxy for nearshore retention, offshore transport, larval recruitment, nutrient availability, metabolic rates, kelp abundance, and phytoplankton concentrations. In the study region, upwelling variability has been shown to control the recruitment of key species in benthic communities, such as barnacles and mussels [10]; this is also true for other comparable locations in Chile and California [37], [49], [50]. Further studies show that the spatial structure of upwelling induces a spatial signal in the recruitment of intertidal invertebrates [14], [51]–[53]. Thus, large-scale changes in upwelling remains as the most likely hypothesis explaining latitudinal changes in recruitment of sessile invertebrates to the rocky intertidal zone [10]. The importance of this spatial pattern is amplified as some of these sessile species can have cascading effects through the benthic community [54].

In addition to being an index of upwelling and recruitment, water temperature can have a significant effect on the vital rates of invertebrates and algae. For instance, temperature has a direct effect on the duration of planktonic larval development in numerous invertebrate species [55]. Temperature changes across a well-documented biogeographic boundary in southern California (Point Conception) are the chief factor explaining spatial shifts in growth rates of intertidal barnacles and mussels [56], [57]. Spatial patterns of temperature have also been related to major changes in the abundance of macroalgal functional groups along the coast of South Africa and Chile [17], as well as on the establishment, germination, and reproduction of intertidal kelps [58]. Temperature is also well correlated with other water properties, including concentration of the limiting nutrient nitrate [32], [59], [60], concentration of dissolved oxygen [61], concentration of carbon dioxide and pH levels [62]. In upwelling areas, nitrate decreases with increasing temperature up to a threshold above which coastal waters are nutrient-depleted [32], [33], [59], [63], typically for temperatures above 14°C in central Chile [32].

In this study we show specifically how the sharp transition in nearshore temperature variability can represent a transition in nearshore nitrate availability, which in turn explains an observed latitudinal change in standing biomass of corticated algae at 30–31°S (Fig. 6). The rare occurrence of sub-14°C waters at the shoreline north of the 30.25°S transition implies limited nitrate supply to nearshore communities; sub-thermocline waters are seldom upwelled and nitrate fluxes are mostly due to vertical mixing. South of the transition, temperature records indicate that nitrate-rich waters are present in the nearshore more than half the time – and this is where higher corticated algal biomass is found (Fig. 6). Clearly, this could be a primary factor in the documented latitudinal discontinuities in functional group abundance [17], genetic structure [19], and range limits of intertidal and shallow-subtidal macroalgae [15], [16], [21], [42], [64]. Further, the thermal transition at 30.25°S may be sufficient to represent different selective environments, particularly for species with limited dispersal, such as kelp [19] or red algae [21]. Recent studies have also documented latitudinal breaks in the reproductive patterns of invertebrates along this region, e.g., significantly fewer embryos per unit area of egg-capsule have been documented for the gastropod *Concholepas concholepas* at sites north of 29–30°S [65]. Our study provides a guide for which hydrographic and dynamic characteristics of the coastal ocean could be tested as the drivers for specific biological changes along this section of the Chilean shore.

## Conclusion

Our findings suggest that in addition to mesoscale variability associated with cape-and-bay coastal topography, a latitudinal discontinuity in nearshore thermal regimes is found at 30.25°S. This marked transition is characterized by sudden shifts in seasonality, synoptic variability, nearshore stratification, and shoreline water temperatures. The localized alongshore change results from the interaction of latitudinal trends (e.g., wind stress, surface warming, inertial period) with a major headland-bay system that juxtaposes a long stretch of coast characterized by upwelling, which is more representative of southern latitudes, with a long stretch of coast characterized by warm surface waters and stratification, which are more representative of northern latitudes. This transition in thermal conditions likely generates a number of latitude-dependent controls on ecological processes in the nearshore that can explain species-specific effects, and add strength to the suggestion of an oceanography-driven, major spatial transition in coastal communities between 30° and 31°S. The extent to which spatial pattern in nearshore ecosystem structure and function is related to the strength and/or persistence of such a break in oceanographic character is just beginning to be elucidated.

## Supporting Information

Figure S1 **Comparison of coastal wind velocities measured at one of the study sites (ECIM, see** **Fig. 1** **) and wind measurements derived from daily QuikSCAT fields.** Meridional wind velocities at ECIM were averaged over 24-h periods prior to the analysis of correlation (A) and coherence/phase (B, C). Dashed horizontal line in (B) corresponds to the 95% significance level.
(EPS)

Figure S2 **Results of Empirical Orthogonal Function (EOF) analysis of** ***in situ*** **temperature series (top panels) and satellite-derived alongshore winds (bottom panels).** Spatial modes (A, D), as well as temporal modes (B, E) and their corresponding spectral diagrams (C, F) are shown for each analysis. The ca. 3-year period shown in (B, E) corresponds to the section of our temperature record that was gap-free for all sites.
(EPS)

Figure S3 **Comparison of** ***in situ*** **temperature records and satellite-derived SST data (MODIS-Aqua, Level-3 daily images) for pixels located 5 km off each site.** All correlation coefficients are significant at α = 0.01.
(EPS)
